# Assessing microscope image focus quality with deep learning

**DOI:** 10.1186/s12859-018-2087-4

**Published:** 2018-03-15

**Authors:** Samuel J. Yang, Marc Berndl, D. Michael Ando, Mariya Barch, Arunachalam Narayanaswamy, Eric Christiansen, Stephan Hoyer, Chris Roat, Jane Hung, Curtis T. Rueden, Asim Shankar, Steven Finkbeiner, Philip Nelson

**Affiliations:** 1 Google Inc, Mountain View, CA USA; 2 Taube/Koret Center for Neurodegenerative Disease Research and DaedalusBio, Gladstone, USA; 3 Imaging Platform, Broad Institute of Harvard and MIT, Cambridge, MA USA; 4 Department of Chemical Engineering, Massachusetts Institute of Technology (MIT), Cambridge, MA USA; 5 Laboratory for Optical and Computational Instrumentation, University of Wisconsin at Madison, Madison, WI USA; 6 Departments of Neurology and Physiology, University of California, San Francisco, CA USA

**Keywords:** Image analysis, Deep learning, Machine learning, Focus, Defocus, Image quality, Open-source, ImageJ, CellProfiler

## Abstract

**Background:**

Large image datasets acquired on automated microscopes typically have some fraction of low quality, out-of-focus images, despite the use of hardware autofocus systems. Identification of these images using automated image analysis with high accuracy is important for obtaining a clean, unbiased image dataset. Complicating this task is the fact that image focus quality is only well-defined in foreground regions of images, and as a result, most previous approaches only enable a computation of the relative difference in quality between two or more images, rather than an absolute measure of quality.

**Results:**

We present a deep neural network model capable of predicting an absolute measure of image focus on a single image in isolation, without any user-specified parameters. The model operates at the image-patch level, and also outputs a measure of prediction certainty, enabling interpretable predictions. The model was trained on only 384 in-focus Hoechst (nuclei) stain images of U2OS cells, which were synthetically defocused to one of 11 absolute defocus levels during training. The trained model can generalize on previously unseen real Hoechst stain images, identifying the absolute image focus to within one defocus level (approximately 3 pixel blur diameter difference) with 95% accuracy. On a simpler binary in/out-of-focus classification task, the trained model outperforms previous approaches on both Hoechst and Phalloidin (actin) stain images (F-scores of 0.89 and 0.86, respectively over 0.84 and 0.83), despite only having been presented Hoechst stain images during training. Lastly, we observe qualitatively that the model generalizes to two additional stains, Hoechst and Tubulin, of an unseen cell type (Human MCF-7) acquired on a different instrument.

**Conclusions:**

Our deep neural network enables classification of out-of-focus microscope images with both higher accuracy and greater precision than previous approaches via interpretable patch-level focus and certainty predictions. The use of synthetically defocused images precludes the need for a manually annotated training dataset. The model also generalizes to different image and cell types. The framework for model training and image prediction is available as a free software library and the pre-trained model is available for immediate use in Fiji (ImageJ) and CellProfiler.

## Background

Acquiring high quality optical microscopy images reliably can be a challenge for biologists, since individual images can be noisy, poorly exposed, out-of-focus, vignetted or unevenly illuminated, or contain dust artifacts. These types of image degradation may occur on only a small fraction of a dataset too large to survey manually, especially in high-content screening applications [1].

One specific area, image focus quality, is particularly challenging to identify in microscopy images. As described in Bray et al. [2], the task of selecting the best-focus image given a focal z-stack of multiple images of the same sample has been previously explored. For a related but different task, Bray et al. [2] evaluated the performance of several focus metrics operating on a set of single-z-depth images (not focal z-stacks), rated by a human as either in or out-of-focus, and identified the power log-log slope (PLLS) metric to be the best at this task. The PLLS metric is computed by plotting the one-dimensional power spectral density of a given image as a function of frequency on a log-log scale, and fitting a line to the resulting plot; the slope of that line (a single scalar) is the PLLS metric for that image. As described in Bray et al. [3], this value is always negative, and is lower in images where defocus blur removes high-frequencies in the image. The separation of in-focus from out-of-focus images in a dataset using the PLLS metric requires a user-selected threshold, making it difficult to interpret the absolute value of the metric on any given image. This requirement of a threshold, likely different for each image channel [3], precludes the possibility of online automated focus quality analysis during image acquisition. Automatic identification of absolute focus quality of a single image in isolation, without any user-supplied, dataset-specific threshold, has remained an unsolved problem.

Recent advances in deep learning have enabled neural networks to achieve human-level accuracy on certain image classification tasks [4]. Such deep learning approaches require minimal human input to use, in terms of hand-engineered features or hand-picked thresholds, have recently been applied to microscopy images of cells as well [5–9]. Though the automatic detection of low quality images in photographic applications has been explored [10], microscope images differ from consumer photographic images in several important ways. Most microscope images are shift and rotation invariant, have varying offset (black-level) and pixel gain, photon noise [11], and a larger (up to 16-bit) dynamic range. In fluorescence microscopy, just one of the various different microscopy imaging modalities, an image may correspond to one of many possible fluorescent markers each labeling a specific morphological feature. Finally, with high resolution microscopy, the much narrower depth-of-field makes it more challenging to achieve a correct focus, and typical microscope hardware autofocus systems will determine focus based on a reference depth which only roughly correlates with the desired focus depth.

To more precisely identify absolute image focus quality issues across image datasets of any size, including single images in isolation, we have trained a deep neural network model to classify microscope images into one of several physically-relatable absolute levels of defocus. Our work here includes several contributions to enable more precise and accurate automatic assessment of microscope focus quality. First, we frame the prediction problem as an ordered multi-class classification task (as opposed to a regression, as in [5]) on image patches, enabling the expression of prediction uncertainty in image patches with no cells or objects as well as a visualization of focus quality within each image. We then show that a deep neural network trained on synthetically defocused fluorescence images of U2OS cells with Hoechst stain [2], can generalize and classify real out-of-focus images of both that same stain and an unseen stain, Phalloidin, with higher accuracy than the previous state-of-the-art PLLS approach. The combination of these two contributions enables the novel ability to predict absolute image focus quality within a single image in isolation. Lastly, we show qualitative results on how our model predictions generalize to an unseen cell type, Human MCF-7 cells, with data from [12].

## Implementation

We first started with a dataset of images consisting of focal stacks (containing both in-focus and multiple out-of-focus images) of U2OS cancer cells with Hoechst stain from Bray et al. [2], for which we later used to train and evaluate a model’s predictive capabilities. These microscope image datasets have several notable properties: the image focus across a given image can vary but is typically locally consistent, many regions of images consist of just the (typically dark) background, for which there exists no notion of focus quality, and the visible image blur scales approximately linearly with distance from the true focal plane. With these considerations, we sought to train a model that could identify, on a small 84 × 84 image patch (about several times the area of a typical cell), both the severity of the image blur and whether the image blur is even well-defined (e.g. if the image patch is just background).

**Fig. 1:**
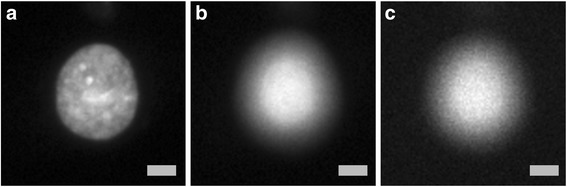
The training data consists of synthetically defocused Hoechst stain images of U2OS cells. **a** A real in-focus image of a cell. **b** A real out-of-focus image of the same cell. **c** A synthetically defocused image, with Poisson noise applied, from the image in (**a**). Scale bars are 10 μm or 15 pixels

**Fig. 2:**
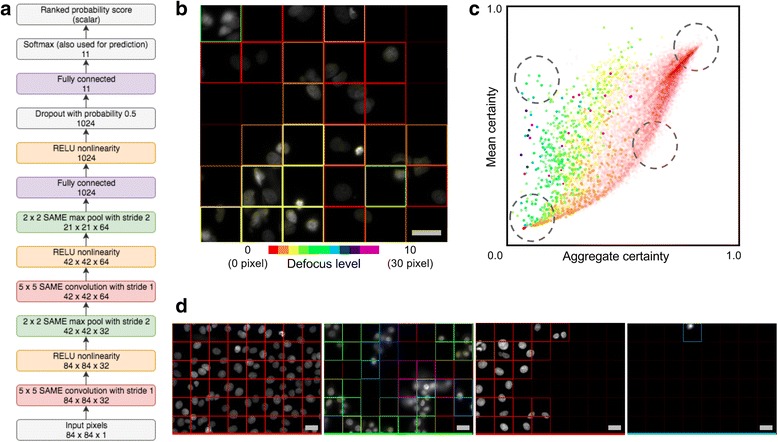
**a** Neural network model architecture; a probability distribution over 11 discrete focus classes is predicted for each input 84 × 84 image patch. This distribution can be summarized (see text) with two scalar values, the predicted defocus level and certainty of that prediction. **b** Example image annotated with patch-level predictions. The patch outlines have one of 11 hues denoting the predicted defocus level and increasing lightness denoting increased certainty. Defocus level ranges from in-focus to out-of-focus with an approximate blur diameter of 30 pixels. **c** A scatter plot of mean versus aggregate certainty, where each point corresponds to one Hoechst stain image of Human MCF-7 cells in the BBBC021 dataset [12], with hue denoting the predicted defocus level as in (**b**). **d** Example images from the circled regions are shown with patch-level annotations ordered from top to bottom. Scale bar is 20 μm or 60 pixels. Images in (**d**) share same color legend as (**b**). Transparency of points in (**c**) varies with number of images

We set aside half of the images (split by site within a well) for evaluation only, and created a training image dataset by taking the 384 most in-focus (the image within each focal stack with the largest standard deviation across all image pixels) images of the U2OS cancer cells with Hoechst stain from the image set BBBC006v1 [2] from the Broad Bioimage Benchmark Collection [12]. This dataset consists of 32 images of each field of view with 2 μm z-spacing, 696 × 520 image size, 2× binning and 20× magnification. We then synthetically defocused the in-focus images by applying a convolution with the following point spread function evaluated by varying z in 2 μm increments [13]

$$ \boldsymbol{h}\left(\boldsymbol{x},\boldsymbol{y},\boldsymbol{z}\right)={\left|\boldsymbol{C}{\int}_{\mathbf{0}}^{\mathbf{1}}{\boldsymbol{J}}_{\mathbf{0}}\left(\boldsymbol{k}\frac{\boldsymbol{NA}}{\boldsymbol{n}}\sqrt{{\boldsymbol{x}}^2+{\boldsymbol{y}}^{\mathbf{2}}}\boldsymbol{\rho} \right)\boldsymbol{\exp}\left(-\frac{\mathbf{1}}{\mathbf{2}}\boldsymbol{jk}{\boldsymbol{\rho}}^{\mathbf{2}}\boldsymbol{z}{\left(\frac{\boldsymbol{NA}}{\boldsymbol{n}}\right)}^{\mathbf{2}}\right)\boldsymbol{\rho} \boldsymbol{d}\boldsymbol{\rho } \right|}^{\mathbf{2}} $$

where J0 is the Bessel function of the first kind, order zero, k = 2, λ = 500 nm is wavelength, NA = 0.5 is numerical aperture, *n* = 1.0 is refractive index and C is a normalization constant. These parameters were our best estimates of the actual imaging parameters, and resulted in image blur diameters from approximately 3 to 30 pixels. We then applied Poisson noise, accounting for image sensor offset and gain. Figure 1 shows an example of such a synthetically defocused image. We trained the model shown in Fig. 2a to predict, for each image patch, a probability distribution over the 11 ordered categories or defocus levels, corresponding to approximately linearly increasing image blur from the perfectly in-focus image (defocus level 0, Fig. 1a). While Fig. 1 shows cell-centered image crops, the actual model was trained on randomly positioned 84 × 84 image crops of the 696 × 520 original size images, many of which contained only the image background and no cells.

We then developed methods to aggregate and visualize the independent predictions on non-overlapping patches within a single image, as well as the set of predictions across a set of images. For each 84 × 84 image patch, the predicted probability distribution or softmax output, {*p*_*i*_} for *i* ∈ {1, …, *N*} for *N* = 11 defocus levels, yields a measure of certainty in the range [0.0, 1.0], computed by normalizing the information entropy of the distribution [14]:

$$ certainty=1-\left({\sum}_{i=1}^N{p}_i\mathit{\log}{p}_i\right)/ logN. $$

Both the most probable class and the prediction certainty can be visualized for each image patch as a colored border, with the hue indicating the predicted class (defocus level) and the lightness denoting the certainty, as shown in Fig. 2b.

The whole-image predicted probability distribution is taken to be the certainty-weighted average of the distributions predicted for the individual patches. The whole-image aggregate certainty is the entropy of that probability distribution. The mean certainty, the average of the individual patch certainties, is plotted against the aggregate certainty in Fig. 2c, for each image in the BBBC021 dataset [12], allowing the identification of several interesting regimes, shown in Fig. 2d (from top to bottom): images with high patch certainty and consistency, images where individual patch certainty is high but the patch predictions are inconsistent, images with only a few high certainty patches, and images with nothing. Importantly, this dataset differed from the training dataset in that it consisted of single z-depth images acquired with 1280 × 1024 image size, 1× binning, 20× magnification and 0.45 NA of Human MCF-7 cells.

To be more precise, a deep neural network was trained on the following image classification task. Given training examples of 16-bit 84 × 84 pixel input image patches and the corresponding degree of defocus (one of 11 discrete classes or defocus levels ordered from least to most defocused), the model predicts the probability distribution over those classes. The model (Fig. 2a) consists of a convolutional layer with 32 filters of size 5 × 5, a 2 × 2 max pool, a convolutional layer with 64 filters of size 5 × 5, a 2 × 2 max pool, a fully connected layer with 1024 units, a dropout layer with probability 0.5, and finally a fully connected layer with 11 units, one for each of the defocus levels.

To correctly penalize model errors on the ordered class categories, the model was trained using a ranked probability score loss function [15] instead of cross-entropy loss, for 1 million steps (about one entire day), using 64 replicas, and a learning rate of 5e-6 with the Adam optimizer [16]. In addition, the model was trained with an augmented training dataset generated by applying a random gain and offset, log-uniform in (0.2, 5.0) and (1, 1000), to each image. We found the data augmentation important (see Results section) for training a model to generalize on new images spanning the large range of of both foreground and background intensities within the 16-bit image dynamic range. The model was implemented and trained with TensorFlow [17].

## Results

### In/out-of-focus classification

**Fig. 3:**
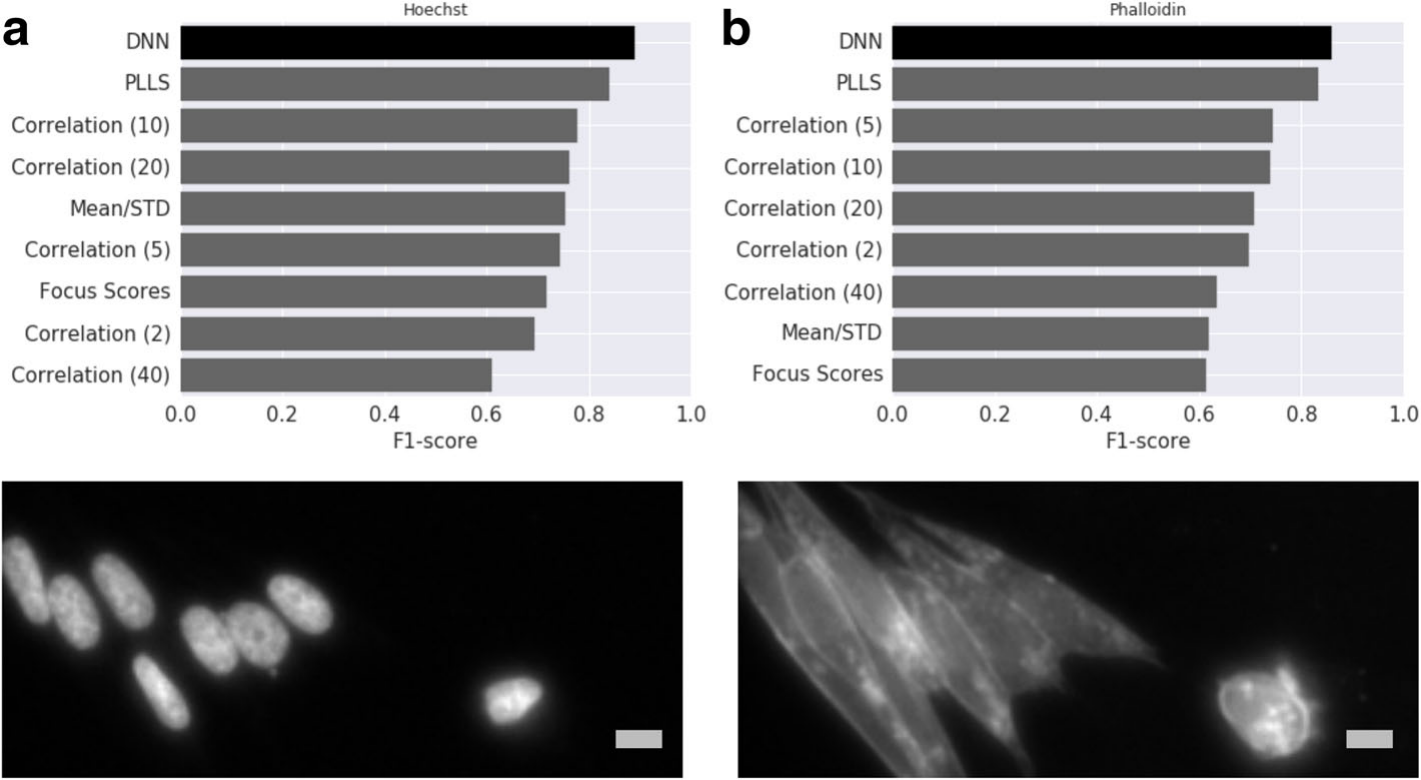
Accuracy, measured with F-score, on the binary in/out-of-focus classification task compared with various methods in Bray et al. [2] for Hoechst (**a**) and Phalloidin (**b**) stained U2OS cell images. The proposed deep neural network (DNN) model (darker bar) trained only on synthetically defocused Hoechst images performs better than the previous approaches evaluated in Bray et al. [2] (lighter bars) on both Hoechst and Phalloidin stain real images, suggesting the model predictions generalize to a qualitatively different unseen stain of the same cell type. Scale bars are 10 μm or 15 pixels

The prediction accuracy was first evaluated on the binary classification task described in Bray et al. [2] on the previously described held out test dataset. This task requires all images be ordered by relative focus quality by some metric, where a user-determined threshold of that metric is used to yield a binary in/out-of-focus prediction for each new image. In Bray et al. [2], several methods in addition to PLLS were evaluated, including Mean/STD, the ratio of average image intensity to standard deviation of image intensity, focus score, a normalized measure of intensity variance within an image, image correlation, evaluated at a particular spatial scale (in pixels). For each metric the optimal user-determined threshold was selected in the following way. Each image in this dataset has a ground truth in-focus or out-of-focus label determined by a human; on a 10% validation subset, the user-determined threshold was selected to maximize the F-score, the harmonic mean of precision and recall, on this subset. Once this threshold has been fixed, it is used to classify each of the remaining 90% test dataset images as in-focus or out-of-focus, and the resulting F-score can be computed and compared with that of other metrics. The model achieved an F-score of 0.89 on the Hoechst stain images, an improvement over the previously reported 0.84 from the PLLS state-of-the-art metric [2] as shown in Fig. 3a.

To assess whether this increase in accuracy might be attributed to our use of a deep neural network model or our framework for aggregating independent quality assessments on smaller image patches, we implemented the PLLS approach using our image patch framework. We first tried to reproduce the previously reported 0.84 f-score, using PLLS on whole images. Due to possible differences in sampling of test and validation images, we observed an f-score of 0.82 instead of 0.84. We then evaluated PLLS with our image patch framework, and observed an F-score of 0.60, suggesting the deep neural network model is responsible for the improved accuracy.

To evaluate the generalization of the model on images representing a novel stain with a qualitatively different appearance from the Hoechst stain training images, the same evaluation procedure in Bray et al. [2] was applied to the Phalloidin (actin) stain images shown in Fig. 3b, yielding an F-score of 0.86, an improvement over the 0.83 achieved by PLLS reported in Bray et al. [2].

Prediction time on each new 2048 × 2048 image was 1.1 s compared with 0.9 s with PLLS, for single-threaded python implementations of each method, and scales linearly with increasing image pixel count.

### Absolute defocus identification

**Fig. 4:**
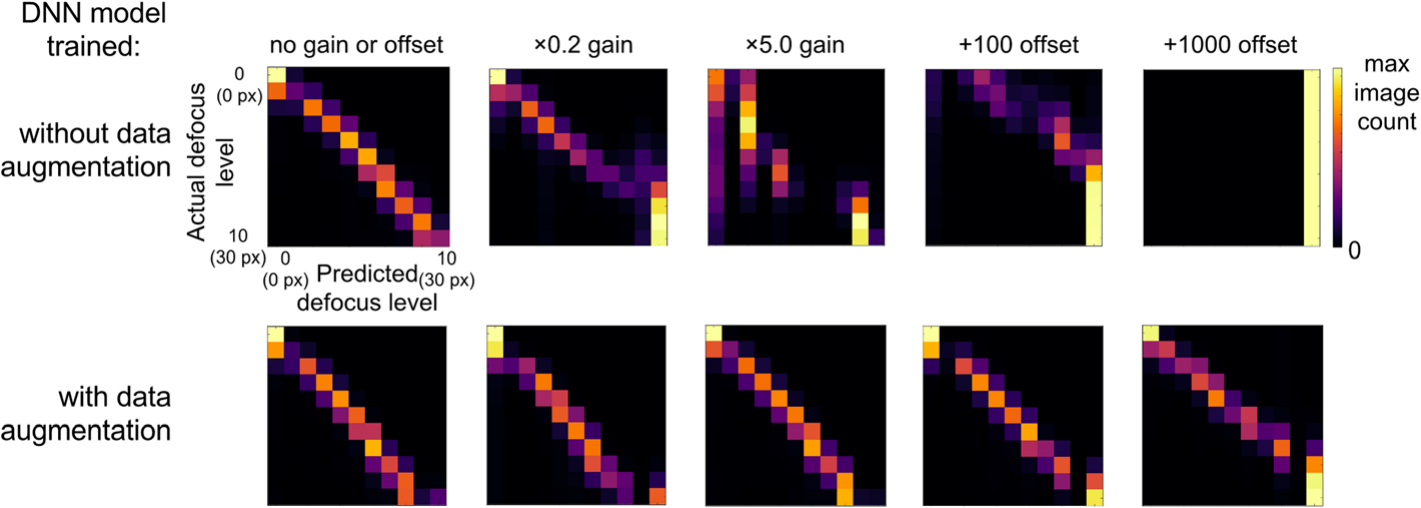
Prediction of absolute focus quality on training data cell type (U2OS cells), Hoechst stain with varying image brightness and background by applying a multiplicative gain and additive offset (16-bit range) to test images. Confusion matrices show the image counts for all pairs of predicted and actual focus levels, where images in each class are separated by a blur diameter of 3 pixels (px). In the absence of a gain or offset (first column), both models perform similarly, but the model trained without data augmentation (first row) is biased toward predicting brighter images as more in-focus, and fails to separate defocus levels entirely with a large offset applied

We next conducted a more fine-grained evaluation using the distance-from-best-focus of the held out image focal stacks in BBBC006 [2] as the ground truth. Here, rather than assess the ability to identify the relative focus of two images after determining an optimal threshold on a validation dataset, we directly assess the ability of the model to identify the absolute defocus level on a single image in isolation, without any user-specified parameters. The lower left confusion matrix in Fig. 4 suggests the model is able to predict within one level of the true defocus level (approximately 3 pixel blur diameter) in 95% of the test images.

To assess the model’s ability to generalize on images with different brightnesses and background offsets, we conducted a test with the same held out ground truth images, except each image was additionally augmented with a gain and offset. The resulting confusion matrix across all 11 classes or defocus levels, for each applied gain or offset, is shown in Fig. 4. When trained without data augmentation (first row), the model appears to be biased in predicting an image to be more defocused if the image has a higher background offset. In contrast, with data augmentation, the model predictions do not appear to be biased by the image and background brightness.

**Fig. 5:**
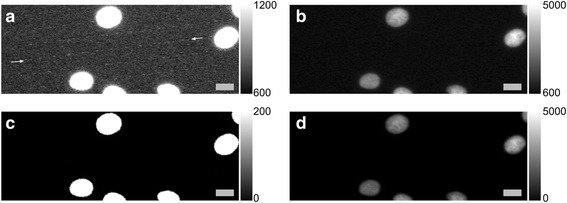
Image artifact removal applied to all images from BBBC021 dataset of MCF-7 cells [12]. Example of a contrast adjusted (**a**) and unadjusted (**b**) original image with noise artifact. The artifact consists of bright pixels oriented along 7 evenly spaced parallel lines with slope of ~ 0.1 (the arrows indicate one such line). The same image, with artifact removal applied as described in main text, shown with contrast adjusted (**c**) and unadjusted (**d**). Scale bar is 10 μm or 30 pixels. Artifact is best viewed in digital form

**Fig. 6:**
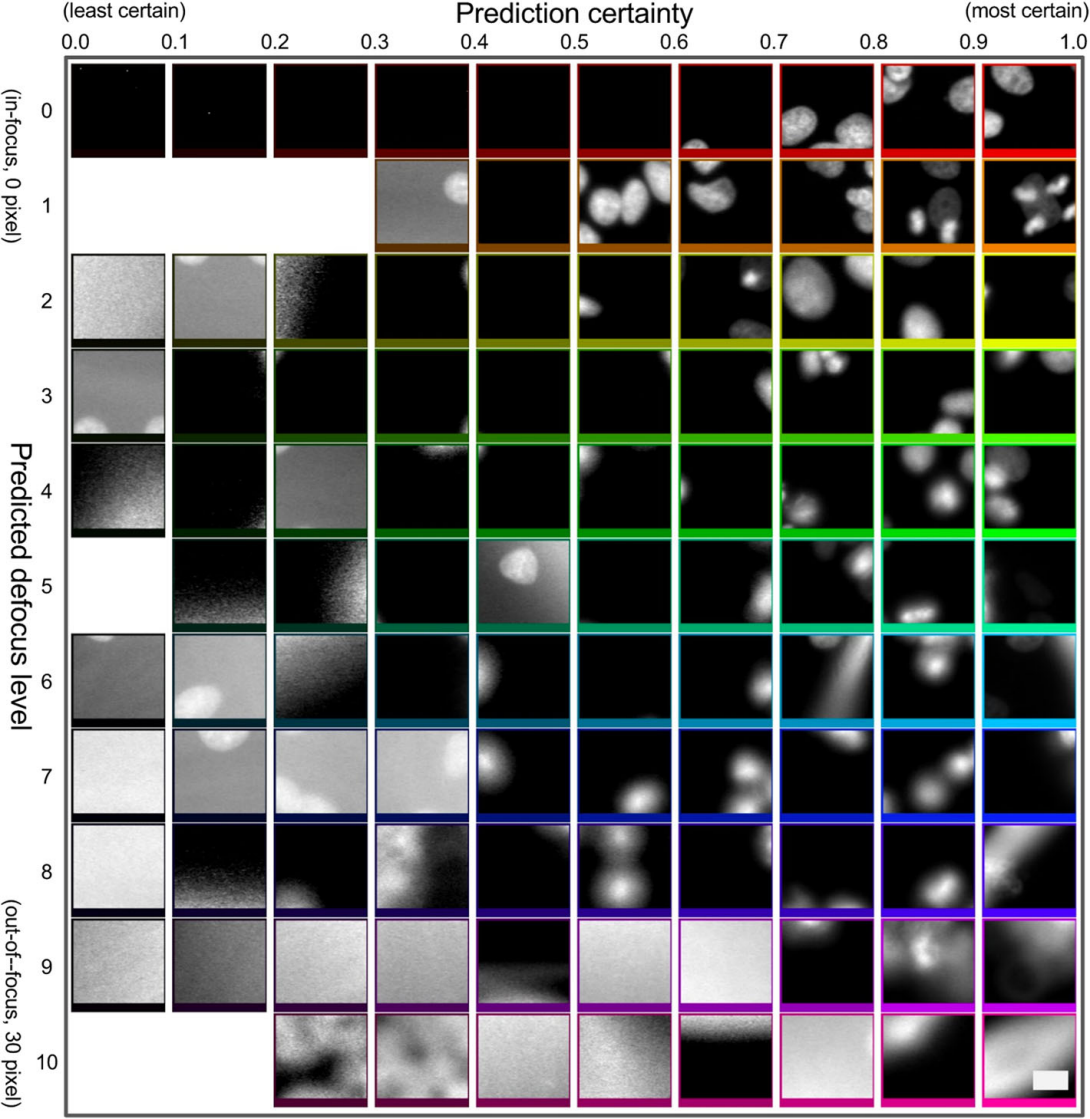
Prediction of absolute focus quality on an unseen cell type (MCF-7 cells, from BBBC021 dataset [12]) but familiar stain, Hoechst. An 11 × 10 image montage showing sample patch-level predictions (for each predicted defocus level (0 for in-focus, 10 for most out-of-focus, corresponding to an approximate blur diameter of 30 pixels) and certainty bin (1.0 is most certain); hue and lightness encode predicted defocus level and certainty, respectively. Blank regions denote combinations of predicted defocus level and certainty for which there are no model predictions for this particular dataset. Scale bar is 10 μm or 30 pixels

**Fig. 7:**
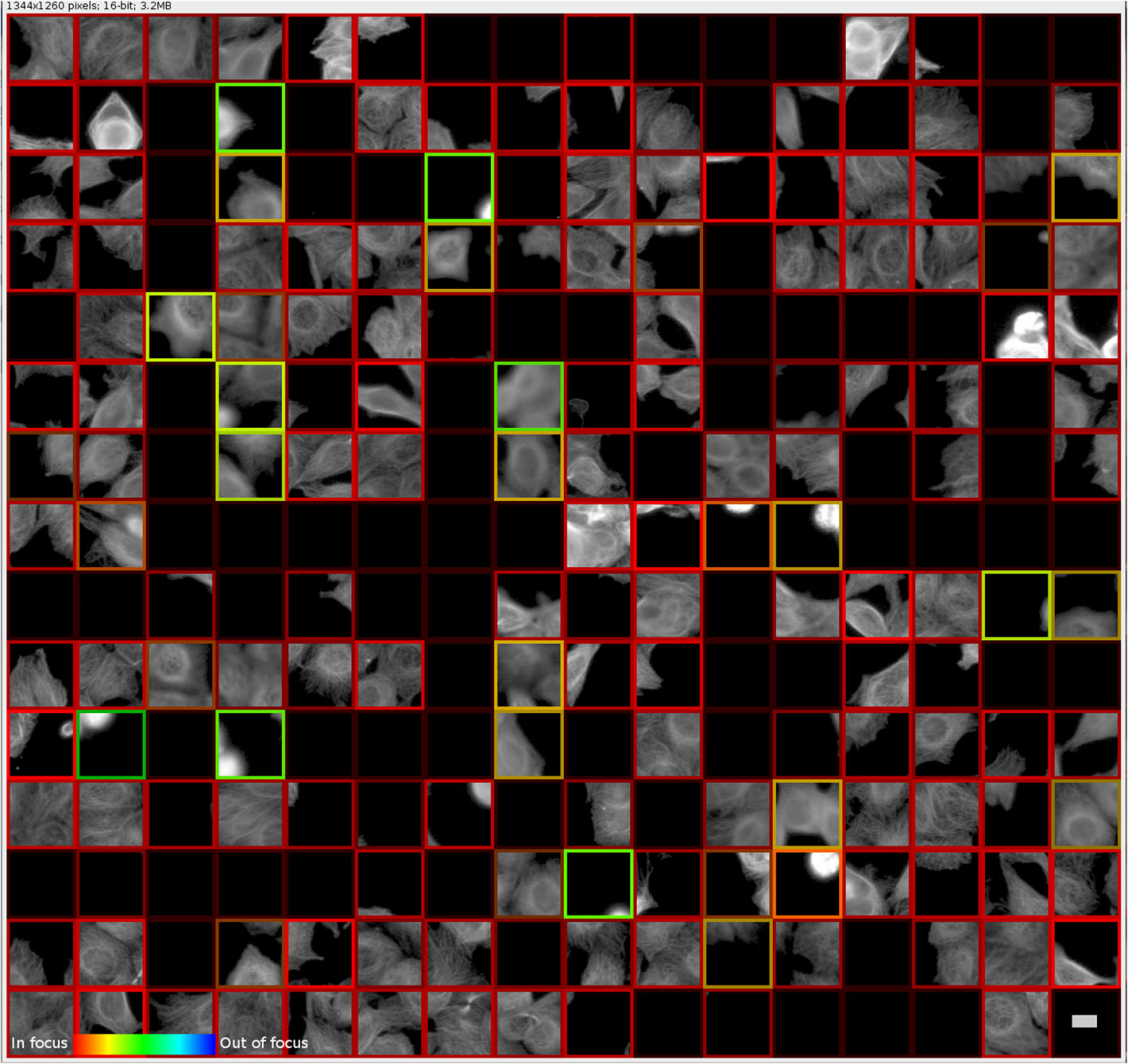
Prediction of absolute focus quality on an unseen cell type (MCF-7 cells, from BBBC021 dataset [12]) and unseen stain, Tubulin, using our Fiji (ImageJ) [20] plugin with pre-trained TensorFlow model. A composite image montage was assembled using the center 84 × 84 patch from a randomly selected batch of 240 images. The border hues denote predicted defocus levels (red for best focus), while the lightness denotes prediction certainty. Scale bar is 10 μm or 30 pixels, and a gamma of 0.45 was applied for viewing

Finally, we conducted a qualitative evaluation of the model on a nominally in-focus dataset consisting of a variety of drug-induced cellular phenotypes of an unseen cell type, Human MCF-7 (BBBC021 [12]). For this dataset only, we observed a subtle image artifact in most images, attributed to a defective camera sensor, shown in Fig. 5, which we removed by subtracting 1000 from every pixel value and clipping the result at zero. Figure 6 shows example predictions on this dataset for Hoechst stain. For the most part, the pre-trained model appears to generalize quite well, though at the image patch level, there are occasionally errors. For example, the patch in predicted defocus level 5, certainty 0.4–0.5 is actually in focus, but with a large background intensity. Lastly, in Fig. 7, we apply the pre-trained model to a montage created with one 84 × 84 image patch from each of 240 Tubulin stain images, where it mostly correctly identifies 3–8% out-of-focus image patches with about 30% background patches.

## Discussion

Rather than train the model on focal stacks of defocused images acquired by a real microscope or manually labeled images, we trained the model on synthetically defocused versions of real images instead. This enabled the use of known ground truth images for training the model to identify absolute focus quality rather than relative measures of quality. Another advantage of this approach is that we can generate the large number of training examples required for deep learning using only an in-focus image dataset, and that the model might be more robust to overfitting on nuisance factors in the experimental data. However, the success of this approach depends on the extent to which the image simulation accurately represents the real physical image formation. We took advantage of the well-known behavior of light propagation in a microscope to achieve this. We note that in certain applications, the use of a more complex optical model may yield even better results [18].

Our analysis demonstrated the importance of using data augmentation to train a model to handle the large range of possible foreground and background intensities. Not only does our learning-based approach enable prediction of an absolute measure of image focus quality on a single image, but it also requires no user-specified parameters for preprocessing the input images.

Possible future work includes training the model to predict on even more varied input images, including those spanning multiple spatial scales, additional imaging modalities such as brightfield, cell types, stains and phenotypes. These extensions might be implemented by a combination of a more accurate image simulator and the inclusion of a more diverse and representative dataset of in-focus real training images. In particular, additional image datasets would enable a more comprehensive assessment of model generalization beyond what has been presented here, and, along with an improved assessment methodology, would allow for a better comparison of the methods compared in [2] and presented in Fig. 3, including statistical significance of accuracy gains, which we did not assess. Optimizing the network size, input image patch dimensions or explicitly modeling background image patches where focus is undefined might improve accuracy further. Lastly, the current model specializes in the task of determining focus quality, but additional measures of image quality could be explored as additional prediction tasks, with simulated data for training.

## Conclusions

A deep learning model was trained on synthetically defocused versions of real in-focus microscope images. The model is able to predict an absolute measure of image focus on a single image in isolation, without any user-specified parameters and operates at the image-patch level, enabling interpretable predictions along with measures of prediction uncertainty. Out-of-focus images are identified more accurately compared with previous approaches and the model generalizes to different image and cell types. The software for training the model and making predictions is open source and the pre-trained model is available for download and use in both Fiji (ImageJ) [19, 20] and CellProfiler [21].

## Abbreviations

DNN: Deep neural network
Fiji: Fiji is just ImageJ
PLLS: Power log-log slope
